# Computed Tomography–Based Biomarker Outcomes in a Prospective Trial of Preoperative FOLFIRINOX and Chemoradiation for Borderline Resectable Pancreatic Cancer

**DOI:** 10.1200/PO.19.00001

**Published:** 2019-08-23

**Authors:** Eugene J. Koay, Matthew H.G. Katz, Huamin Wang, Xuemei Wang, Laura Prakash, Milind Javle, Rachna Shroff, David Fogelman, Santiago Avila, Mohamed Zaid, Dalia Elganainy, Yeonju Lee, Christopher H. Crane, Sunil Krishnan, Prajnan Das, Jason B. Fleming, Jeffrey E. Lee, Eric P. Tamm, Priya Bhosale, Jeffrey H. Lee, Brian Weston, Anirban Maitra, Robert A. Wolff, Gauri R. Varadhachary

**Affiliations:** ^1^ University of Texas MD Anderson Cancer Center, Houston, TX; ^2^ Memorial Sloan Kettering Cancer Center, New York, NY; ^3^ Moffitt Cancer Center, Tampa Bay, FL

## Abstract

**PURPOSE:**

Effective preoperative regimens and biomarkers for pancreatic ductal adenocarcinoma (PDAC) are lacking. We prospectively evaluated fluorouracil, leucovorin, irinotecan, and oxaliplatin (FOLFIRINOX)-based treatment and imaging-based biomarkers for borderline resectable PDAC.

**METHODS:**

Eligible patients had treatment-naïve, histology-confirmed PDAC and one or more high-risk features: mesenteric vessel involvement, CA 19-9 level of 500 mg/dL or greater, and indeterminate metastatic lesions. Patients received modified FOLFIRINOX and chemoradiation before anticipated pancreatectomy. Tumors were classified on baseline computed tomography as high delta (well-defined interface with parenchyma) or low delta (ill-defined interface). We designated computed tomography interface response after therapy as type I (remained or became well defined) or type II (became ill defined). The study had 80% power to differentiate a 60% from 40% resection rate (α = .10). Overall survival (OS) and progression-free survival (PFS) were estimated using the Kaplan-Meier method, and subgroups were compared using log-rank tests.

**RESULTS:**

Thirty-three patients initiated therapy; 45% underwent pancreatectomy. The median OS was 24 months (95% CI, 16.2 to 29.6 months). For patients who did and did not undergo pancreatectomy, the median OS was 42 months (95% CI, 17.7 months to not estimable) and 14 months (95% CI, 9.0 to 24.8 months), respectively. Patients with high-delta tumors had lower 3-year PFS (4% *v* 40%) and 3-year OS rates (20% *v* 60%) than those with low-delta tumors (both *P* < .05). Patients with type II interface responses had lower 3-year PFS (0% *v* 29%) and 3-year OS rates (16% *v* 47%) than those with type I responses (both *P* < .001).

**CONCLUSION:**

Preoperative FOLFIRINOX followed by chemoradiation for high-risk borderline resectable PDAC was associated with a resection rate of 45% and median OS of approximately 2 years. Our imaging-based biomarker validation indicates that personalized treatment may be achieved using these biomarkers at baseline and post-treatment.

## INTRODUCTION

Personalized treatment of patients with pancreatic ductal adenocarcinoma (PDAC) has been limited by the dearth of validated biomarkers. Clinicians have prognostic biomarkers but lack predictive markers to match appropriate treatments to each patient. CA 19-9 is the only Food and Drug Administration–approved prognostic biomarker for PDAC, but it is limited to patients with Sialyl Lewis a–positive genotype. Interpretation of this test can be unreliable.^1,2^ *SMAD4*,^3^ which transduces intracellular signaling of transforming growth factor beta, and human equilibrative nucleoside transporter 1^4-6^ may be useful in guiding therapy,^3,7^ but prospective validation has been unsuccessful, highlighting the need for robust biomarker integration for PDAC clinical trials. In a disease that is at-large systemically disseminated on presentation with a propensity for early progression while receiving therapy, there is an unmet need for predictive biomarkers for PDAC that indicate benefit from local therapies, such as radiation and surgical resection.

We have identified CT-based biomarkers using morphologic characteristics of PDAC,^8,9^ each identifying distinct prognostic groups. In a properly designed trial, these imaging-based biomarkers may help predict benefit from surgical resection. At baseline, on a standard-of-care pancreas protocol CT, high-delta PDAC tumors exhibit an abrupt change—or delta—in Hounsfield units (HU) between the visualized tumor and normal pancreas, and low-delta PDAC tumors do not exhibit such a change. After preoperative therapy, tumors with a type I response remain or become well defined at the interface of tumor and parenchyma, whereas those with a type II response become less defined at the interface. Notably, these CT-based biomarkers associate with pathologic features of PDAC, such as the extent of stromal reaction^10^ and pathologic response to therapy.^9^

**Context**

**Key Objective**
Few effective therapies exist for pancreatic ductal adenocarcinoma (PDAC), and no biomarkers have been validated to personalize therapy for this aggressive disease. We report a phase II clinical trial of preoperative modified fluorouracil, leucovorin, irinotecan, and oxaliplatin (FOLFIRINOX) and chemoradiation for patients with high-risk borderline resectable PDAC, powered to differentiate a resection rate of 60% from 40%. We examined whether computed tomography (CT)-based biomarkers had prognostic association with outcomes.
**Knowledge Generated**
The preoperative regimen achieved a resection rate of 45%, lower than the desired 60% rate in this high-risk population. Notably, both a baseline CT-based biomarker (delta classification) and post-treatment CT-based biomarker (interface response) were associated with outcomes. In retrospect, the trial was enriched for patients with poor prognostic biomarkers.
**Relevance**
The validation of biomarkers derived from standard-of-care CT imaging supports the development of trials that use these as integral biomarkers and may lead to personalized therapeutic management for localized PDAC.

We prospectively evaluated clinical associations of CT-based delta scores and CT-interface responses in patients who received preoperative modified fluorouracil, leucovorin, irinotecan, and oxaliplatin (FOLFIRINOX) and gemcitabine-based chemoradiation for localized cancers at high risk for early metastatic progression. The primary objective was rate of resection after preoperative therapy, and secondary objectives included toxicity rates, overall survival (OS), progression-free survival, and correlation with the imaging-based biomarkers.

## METHODS

### Patient Eligibility and Disease Staging

The institutional review board at the University of Texas MD Anderson Cancer Center approved the study protocol (ClinicalTrials.gov identifier: NCT01560949), and all patients provided written informed consent. Enrolled patients were required to have a newly diagnosed, histology-confirmed PDAC and one or more of the following clinical features: a computed tomography (CT) scan of the abdomen using a pancreatic protocol showing a primary tumor associated with deformity of the superior mesenteric vein (SMV) or segmental venous occlusion with a patent vessel above and below suitable for reconstruction; an interface with major arteries that would make pancreatectomy more complex, including the superior mesenteric artery (SMA), celiac artery measuring less than or equal to 180° of the artery’s circumference, and/or the common hepatic artery^11^; a serum CA 19-9 level of 500 mg/dL or more in the presence of a bilirubin level of 2.0 mg/dL or less; and radiographic findings consistent with malignant peripancreatic lymphadenopathy outside the planned radiation or surgical field or liver or peritoneal lesions concerning but not diagnostic for metastatic disease.

Patients were also required to have an Eastern Cooperative Oncology Group performance status (PS) of 0 to 1,^12^ an absolute neutrophil count of more than 1,500 cells/mm^3^, a platelet count of at least 100,000 cells/mm^3^, a serum creatinine level less than 2 mg/dL, a serum bilirubin level less than 2 mg/dL, and hepatic transaminases less than five times the upper limits of normal. When necessary, biliary decompression was accomplished endoscopically by placement of a metal biliary stent. Comorbidity was prospectively measured using the Adult Comorbidity Evaluation 27 index. The severity of each patient’s comorbidity profile was graded as 0 (none), 1 (mild), 2 (moderate), or 3 (severe).^13^

### Treatment Plan

**FIG A1.**
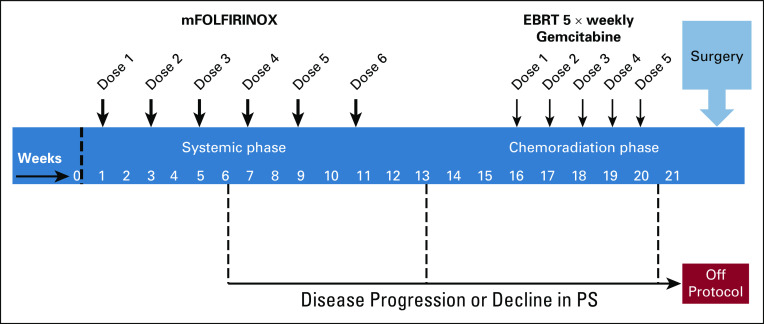
Protocol treatment.

The treatment schema is illustrated in Appendix Figure A1.

#### Preoperative therapy.

Modified FOLFIRINOX, consisting of oxaliplatin (75 mg/m^2^) delivered over a 2-hour period, followed by irinotecan (150 mg/m^2^) given over a 90-minute period, and a continuous infusion of fluorouracil (2,000 mg/m^2^) over 46 hours, was administered once every two weeks for a total of six doses. Modified FOLFIRINOX without a fluorouracil bolus, without leucovorin, and with attenuated doses of oxaliplatin and irinotecan has been reported to show an improved safety profile without compromising efficacy in metastatic PDAC.^14-17^

Within 4 to 6 weeks after completion of modified FOLFIRINOX, patients underwent restaging imaging studies with a pancreas protocol CT scan or magnetic resonance imaging (MRI) scan.^18^ Subsequently, chemoradiation therapy was administered to patients with a PS of 0 or 1 and without evidence of distant progression. 3D conformal radiation therapy at a total dose of 50.4 Gy in 28 fractions (1.8 Gy/fraction) to the gross tumor volume plus the 1.5- to 2-cm margin was prescribed. Radiation was administered concurrently with gemcitabine (350 mg/m^2^) given over 35 minutes once every week for five doses.^19^

#### Surgery.

Restaging with CT or MRI was performed at least 4 to 6 weeks after the last dose of gemcitabine. Patients without local progression or distant metastasis and a PS of 0 or 1 underwent pancreatectomy using standard techniques and typically preceded by staging laparoscopy.^20^ Patients who underwent successful resection on protocol were not offered additional therapy in the adjuvant setting.

### Assessments

#### Radiographic.

All imaging studies for each patient were re-reviewed for tumor-vessel interface (≤ 180° or > 180°, as applicable before surgery) and disease burden by a faculty GI radiologist (P.B.) who was blinded to the clinical history. Response and progression were evaluated using RECIST (Response Evaluation Criteria in Solid Tumor; version 1.1) guidelines.^21^

#### Pathologic.

Analysis of surgical specimens was conducted following the College of American Pathologist guidelines. The pathologic stage was determined using American Joint Committee on Cancer (8th edition) staging.^22^ The specimen was designated as R0 if no tumor cells were identified at any of the resection margins and as R1 if cancer cells were present at the inked bile duct or pancreatic parenchymal margin or at or within 1 mm of the inked SMA margin.^23^

#### Adverse events.

Adverse events during preoperative therapy were recorded using the Common Terminology Criteria for Adverse Events (version 4.0).^24^ Adverse events that occurred within 90 days after surgery were graded using the modified Accordion system.^25^ Severe adverse events were defined as those graded 3 or higher on a 6-point scale. In addition, delayed gastric emptying and postpancreatectomy hemorrhage were graded using International Study Group of Pancreatic Surgery definitions.^26,27^

### Follow-Up

Patients were evaluated every 4 months after treatment. All visits included a history and physical examination, laboratory studies, CT or MRI of the abdomen, and chest x-ray. The development of any new lesion after therapy with characteristics of local relapse or metastatic disease was considered recurrence.

### Correlative Studies

#### Delta classification.

We classified the baseline PDAC morphology according to previously published methods using pretherapy CT images.^8^

#### Interface response.

Patients were evaluated for interface response after chemotherapy and after chemoradiation.^8,9^

### Statistical Analysis

Data were locked for analysis on August 8, 2018. The primary end point of the study was resection rate, defined as the proportion of participants who underwent pancreatectomy among all enrolled participants. Secondary clinical end points included R0 resection rate, toxicity rates, progression-free survival (date of diagnosis to date of disease progression, date of recurrence, or date of death, whichever came first), OS (date of diagnosis to date of death), and patterns of local and distant failure. Additional assessments included associations of the delta classification and interface response with survival.

Patient demographic and clinical characteristics were summarized using median (range) for continuous variables and frequency (percentage) for categorical variables. The associations between binary variables, such as between delta classification or margin status and interface response, were presented using bar plots and were assessed for significance using Fisher’s exact test. The probabilities of OS and PFS were estimated using the Kaplan-Meier method.^28^ Log-rank tests were used to assess the differences in OS or PFS between subgroups of patients defined by delta classification, interface response, and clinical factors (JMP, SAS Institute, Cary, NC). *P* values less than .05 were deemed statistically significant. Adverse events were summarized by frequency, grade, and event type.

The Simon’s optimum two-stage design was applied for this study.^29^ A sample size of 33 was chosen to differentiate between a good resectability rate of 60% and a poor resectability rate of 40% with 80% power and at a significance level of .10. The trial would have been stopped early if seven or fewer patients underwent surgery among the first 16 patients. By the end of the trial, lack of efficacy would have been claimed if 16 or fewer patients underwent surgery among the total 33 enrolled patients.

## RESULTS

### Patients

**FIG 1.**
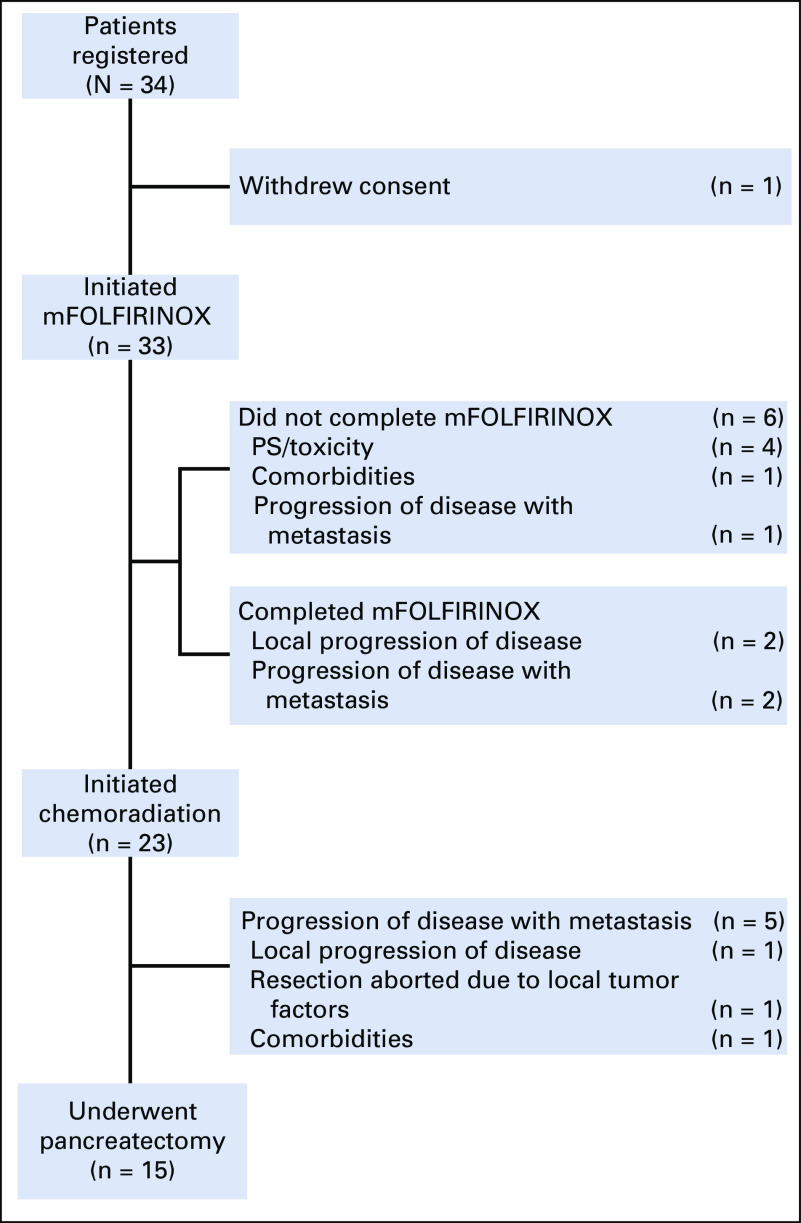
Flow of patients through the protocol treatment. mFOLFIRINOX, modified fluorouracil, irinotecan, leucovorin, and oxaliplatin; PS, performance status

**FIG 2.**
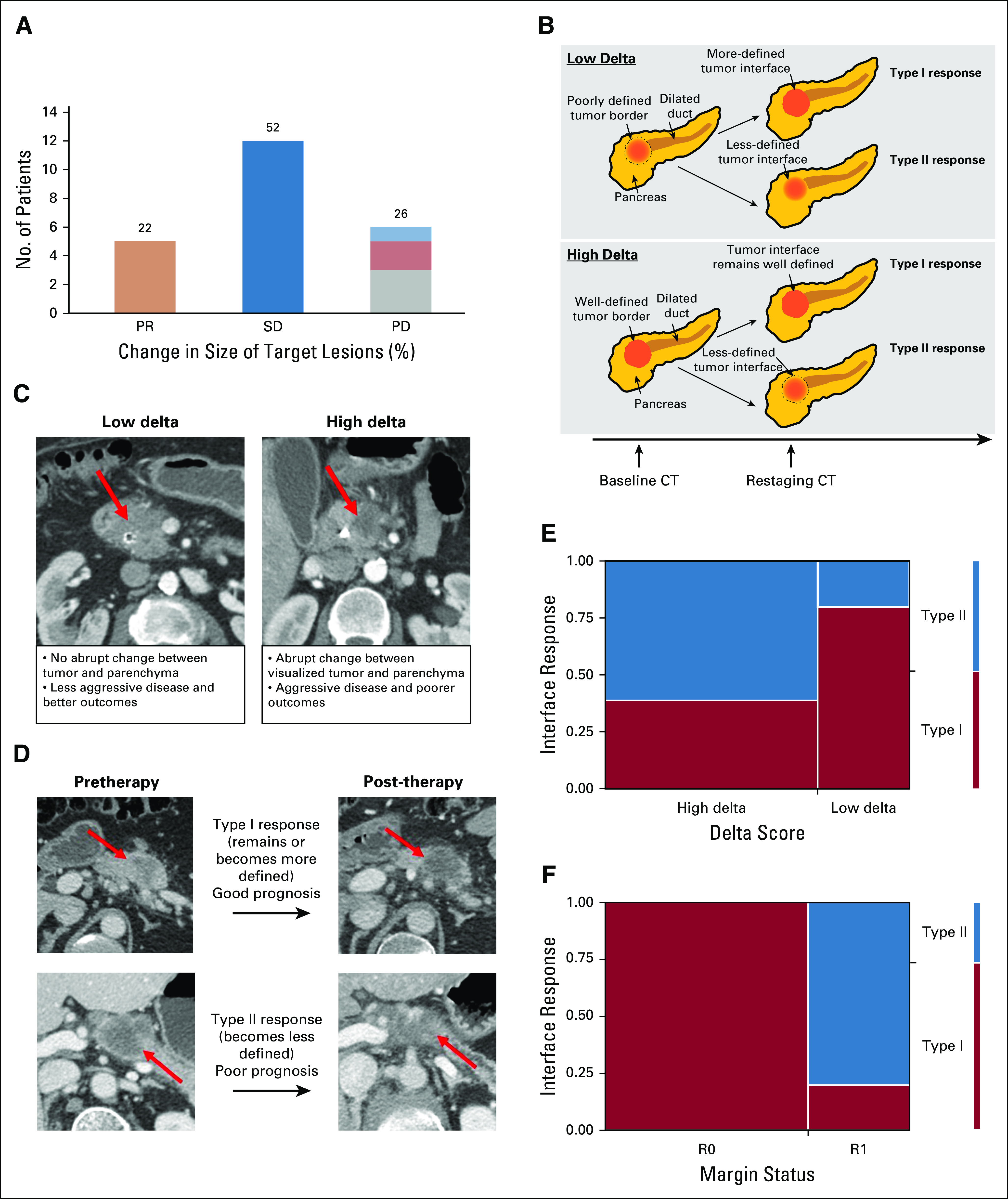
(A) Percent change in size of target lesions for each patient who finished therapy on protocol and had images available for re-review at restaging on the basis of RECIST (Response Evaluation Criteria in Solid Tumor; n = 23). Within the progressive disease (PD) group, one patient had local progression (light blue), three had distant progression (gray), and two had local and distant progression (light red). (B) Schematic illustrating interface response in low-delta and high-delta tumors. (C) Delta classification from computed tomography (CT) scans, showing a low-delta tumor without a distinct interface (arrow, left) and a high-delta tumor with a distinct interface (arrow, right). We applied the same 40 HU cutoff to define low- and high-delta tumors, as described in our previous work.^8^ (D) CT scans showing a type I interface response (top) and a type II interface response (bottom). The interface response is a visual classification whereby type I responses demonstrate that the tumor/pancreas interface remains or becomes well defined, whereas type II responses show the interface becomes less defined after treatment.^9^ Arterial and portal venous phases of the pancreas protocol CT scan are used for the delta and interface response metrics. (E) Association between delta classification and interface response (*P* = .03). (F) Association between margin status and interface response (*P* < .001). PR, partial response; SD, stable disease.

From August 2012 through November 2015, 34 patients enrolled in the study. One patient withdrew consent before initiating treatment. The baseline characteristics of the 33 patients who initiated therapy are listed in Table 1. The median CA 19-9 was 200 U/mL (range, < 1 to 4,112 U/mL), and 11 patients (33%) had a comorbidity profile graded as moderate or severe. The tumor of 14 patients (42%) had a radiographic interface with the SMV–portal vein, and that of 16 (48%) had a radiographic interface between both the SMV–portal vein as well as with the SMA, celiac trunk, or the hepatic artery. The flow of all 33 patients through the treatment protocol is shown in Figure 1, and radiographic responses are shown in Figure 2A.

**TABLE 1.**
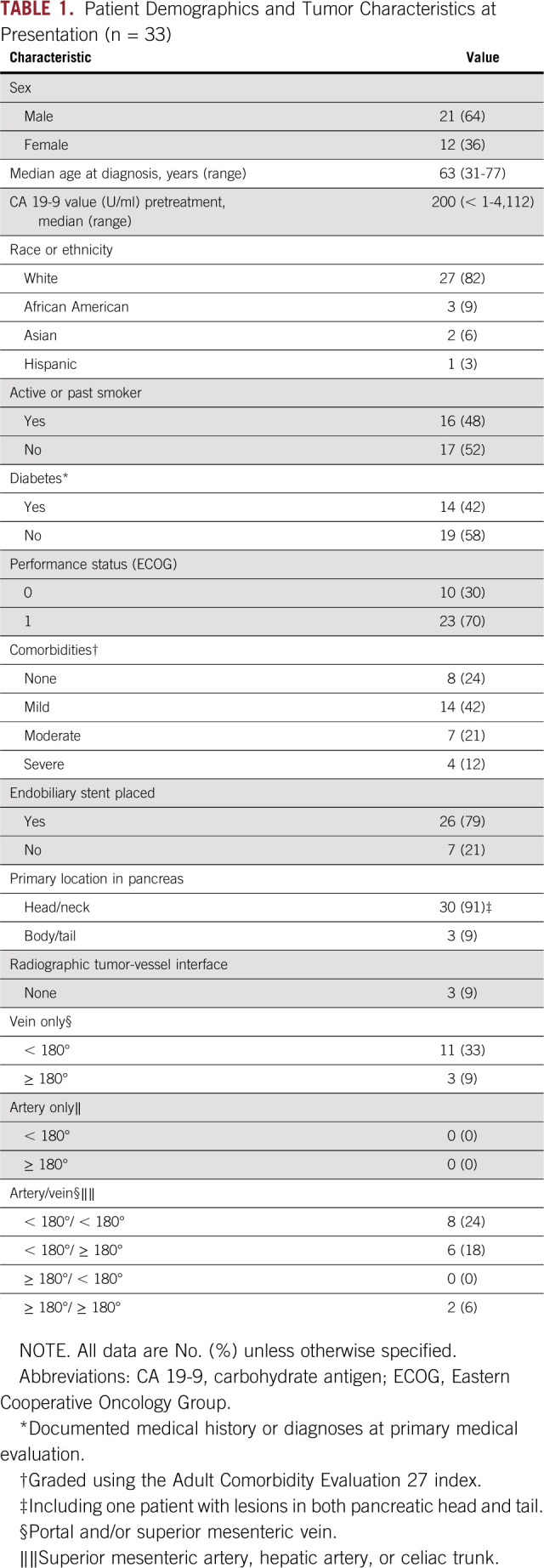
Patient Demographics and Tumor Characteristics at Presentation (n = 33)

### Adverse Events

#### Preoperative therapy.

Of the enrolled patients, 70% completed all six doses of modified FOLFIRINOX. Sixty-four percent of patients received at least four gemcitabine doses during the chemoradiation phase. Table 2 lists the adverse events occurring in more than 5% of patients.

**TABLE 2.**
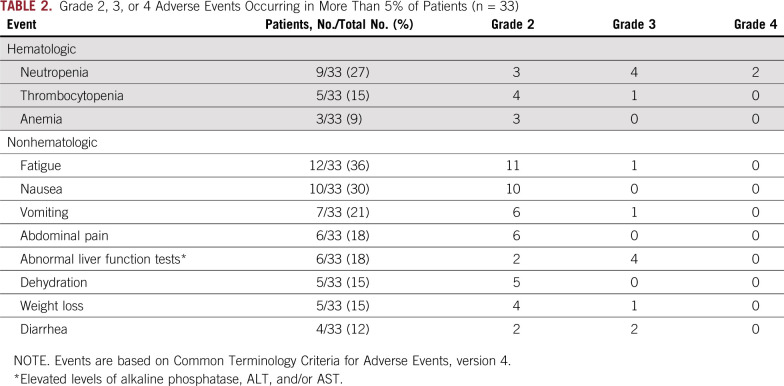
Grade 2, 3, or 4 Adverse Events Occurring in More Than 5% of Patients (n = 33)

#### Pancreatectomy.

Major adverse events within 90 days of pancreatectomy occurred in three of the 15 patients (20%) who underwent pancreatectomy, including postpancreatectomy hemorrhage of grade B (n = 1) and grade C (n = 1) and delayed gastric emptying of grade C (n = 2). There was no perioperative (90-day) mortality (Appendix Table A1).

### Pathologic Response and Margins

The histopathologic characteristics of the 15 resected tumors are listed in Table 3. Ten of the 15 specimens (67%) had negative (R0) surgical margins; cancer cells were identified at or within 1 mm of the SMA margin in four patients and at the SMA and bile duct margin in one patient. Metastatic disease was identified in regional lymph nodes in eight specimens (53%). None of the 15 resected specimens had less than 5% viable cancer cells.

**TABLE 3.**
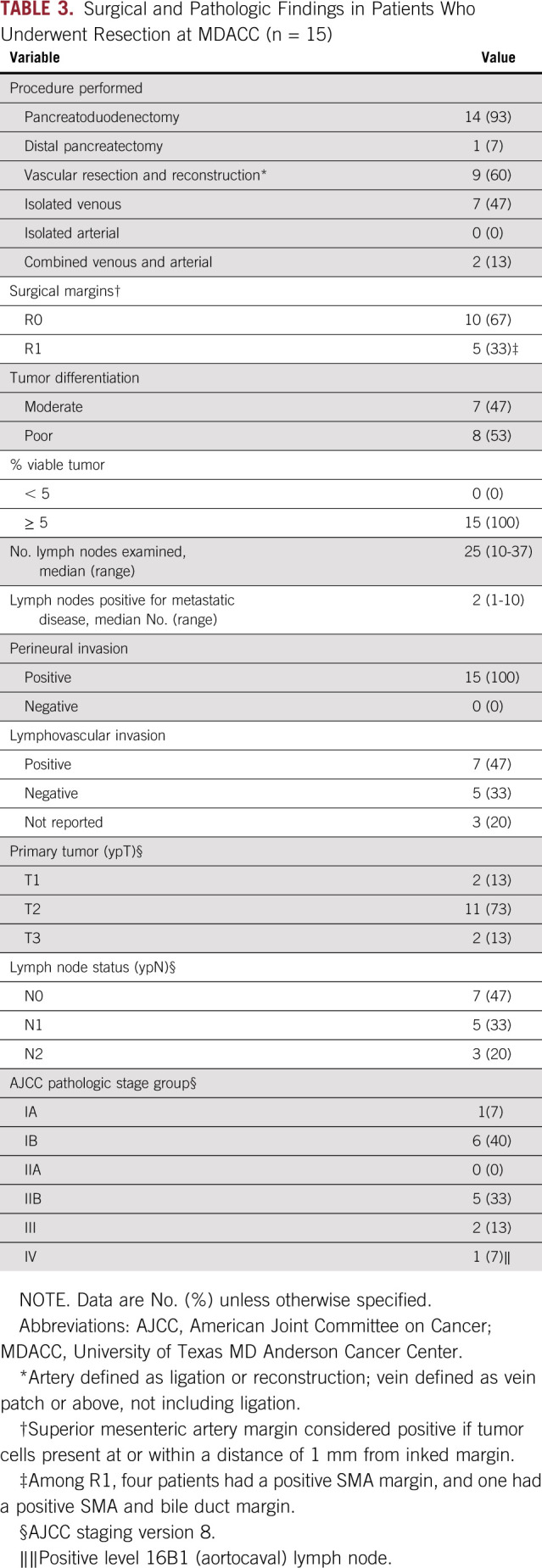
Surgical and Pathologic Findings in Patients Who Underwent Resection at MDACC (n = 15)

### Imaging Biomarkers

#### CT-based delta classification.

**FIG A2.**
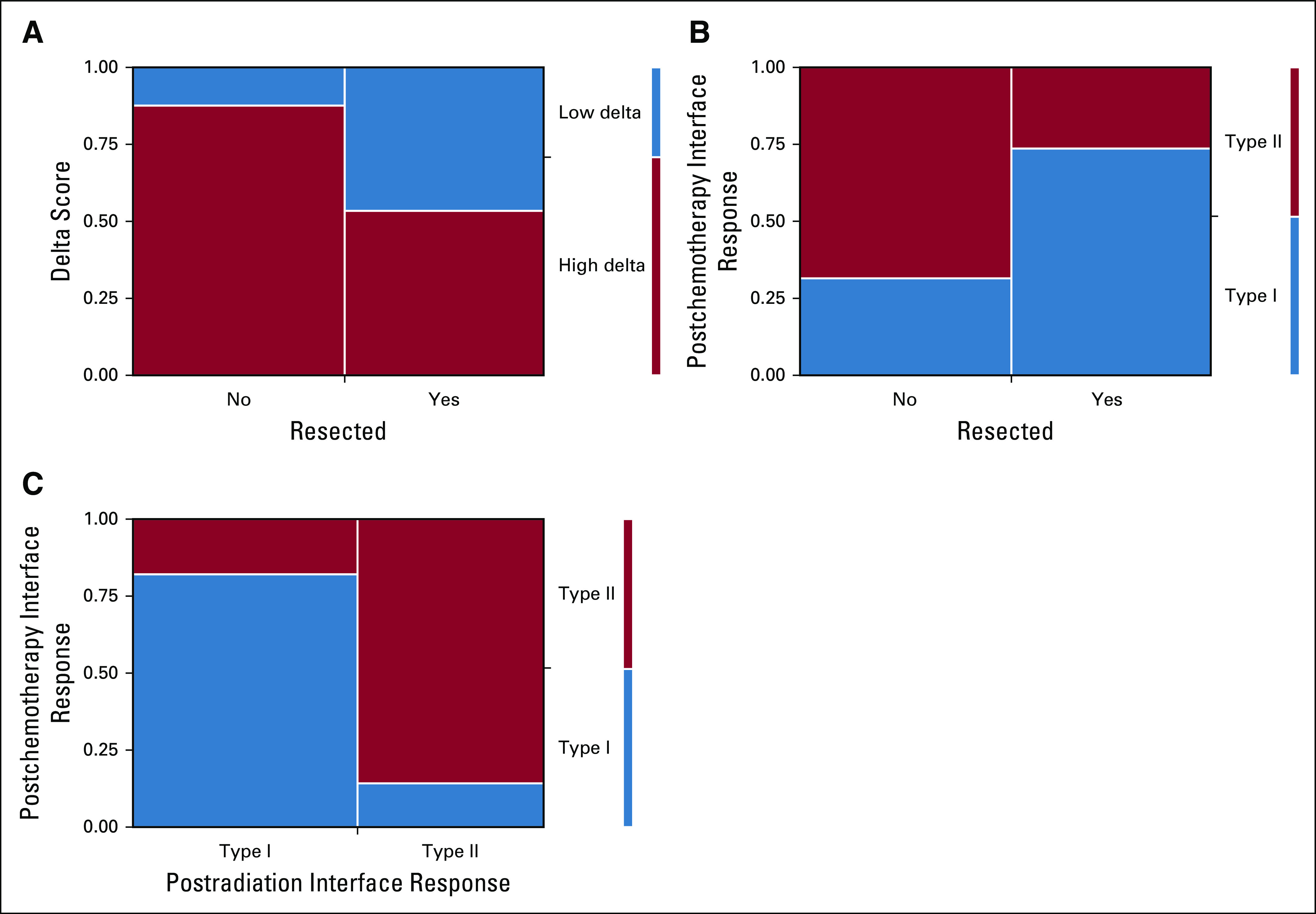
(A)**** Association between delta score and pancreatectomy (*P* = .0326).**** (B) Association between postchemotherapy interface response and pancreatectomy**** (*P* = .0173). (C) Association between interface response after chemotherapy and after radiation therapy (*P* = .0001).

Ten patients had low-delta PDAC, and 23 had high-delta PDAC. High-delta tumors were associated with a lower rate of resection (Figs 2B and 2C; Appendix Fig A2).

#### Interface response.

**FIG A3.**
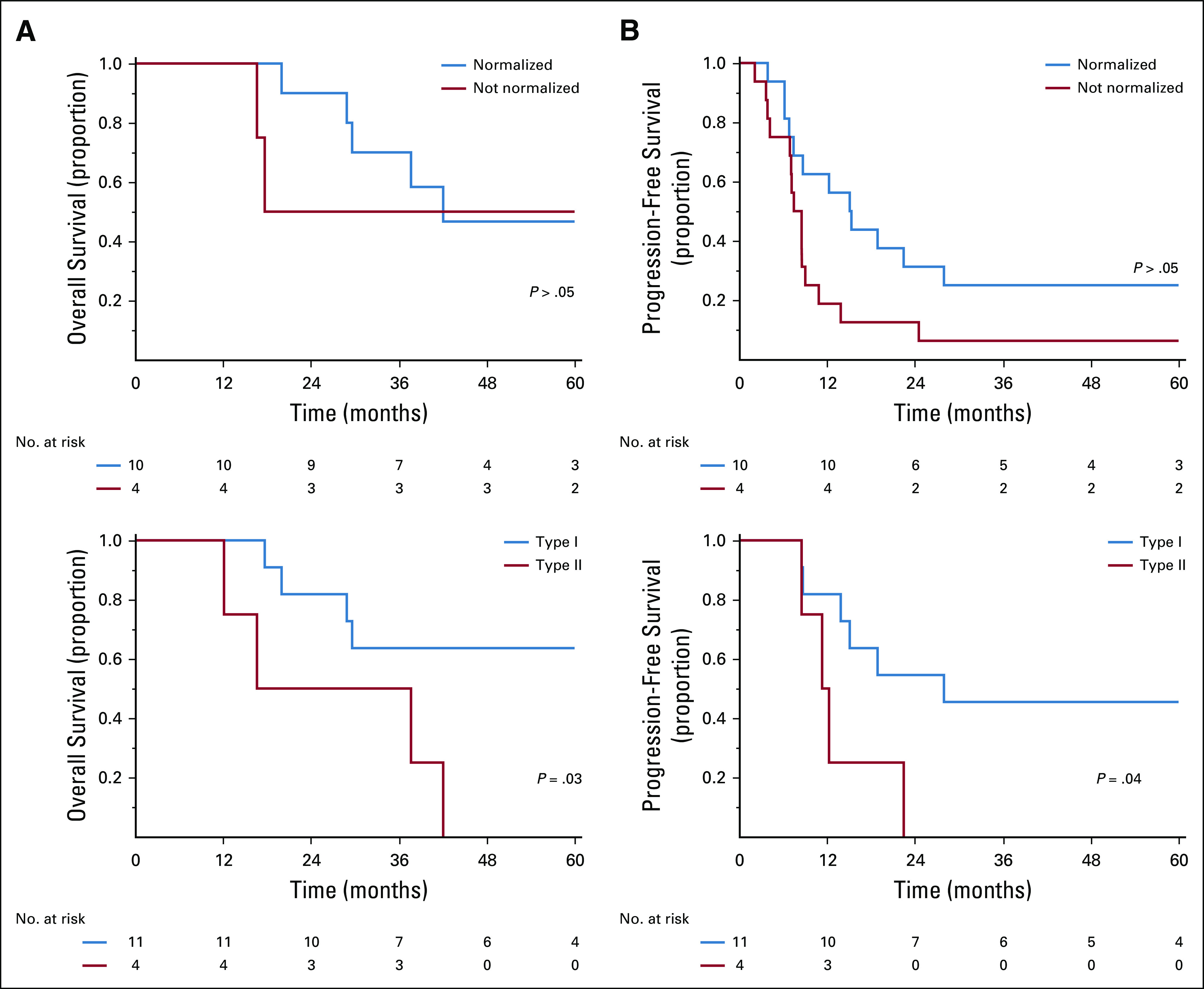
Kaplan-Meier estimates for (A) overall survival and (B) progression-free survival for normalized and not normalized CA 19-9 levels in patients who underwent resection as well as (C) overall survival and (D) progression-free survival by interface response type in patients who underwent resection.

Seventeen patients exhibited a type I response, whereas 16 exhibited a type II response after chemotherapy. Patients who exhibited a type II interface response were less likely to undergo pancreatectomy compared with those who exhibited a type I response (Figs 2B and 2D; Appendix Fig A2). Interface response postchemotherapy was highly associated with interface response postchemoradiation (Appendix Fig A3). Patients with high-delta PDAC were more likely to exhibit a type II interface response compared with those with low-delta PDAC (Fig 2E; *P* = .026). Patients with a type II interface response were more likely to have an R1 resection margin compared with those who exhibited a type I response (Fig 2F; *P* < .001).

### Survival Outcomes

At last follow-up, 24 of 33 patients (73%) died as a result of PDAC: 16 of the 18 patients (89%) who did not undergo surgery on protocol and eight of the 15 patients (53%) who did. Among the 15 patients who underwent pancreatectomy, 10 (67%) had a distant recurrence at last follow-up. There were no instances of isolated local recurrence.

**FIG 3.**
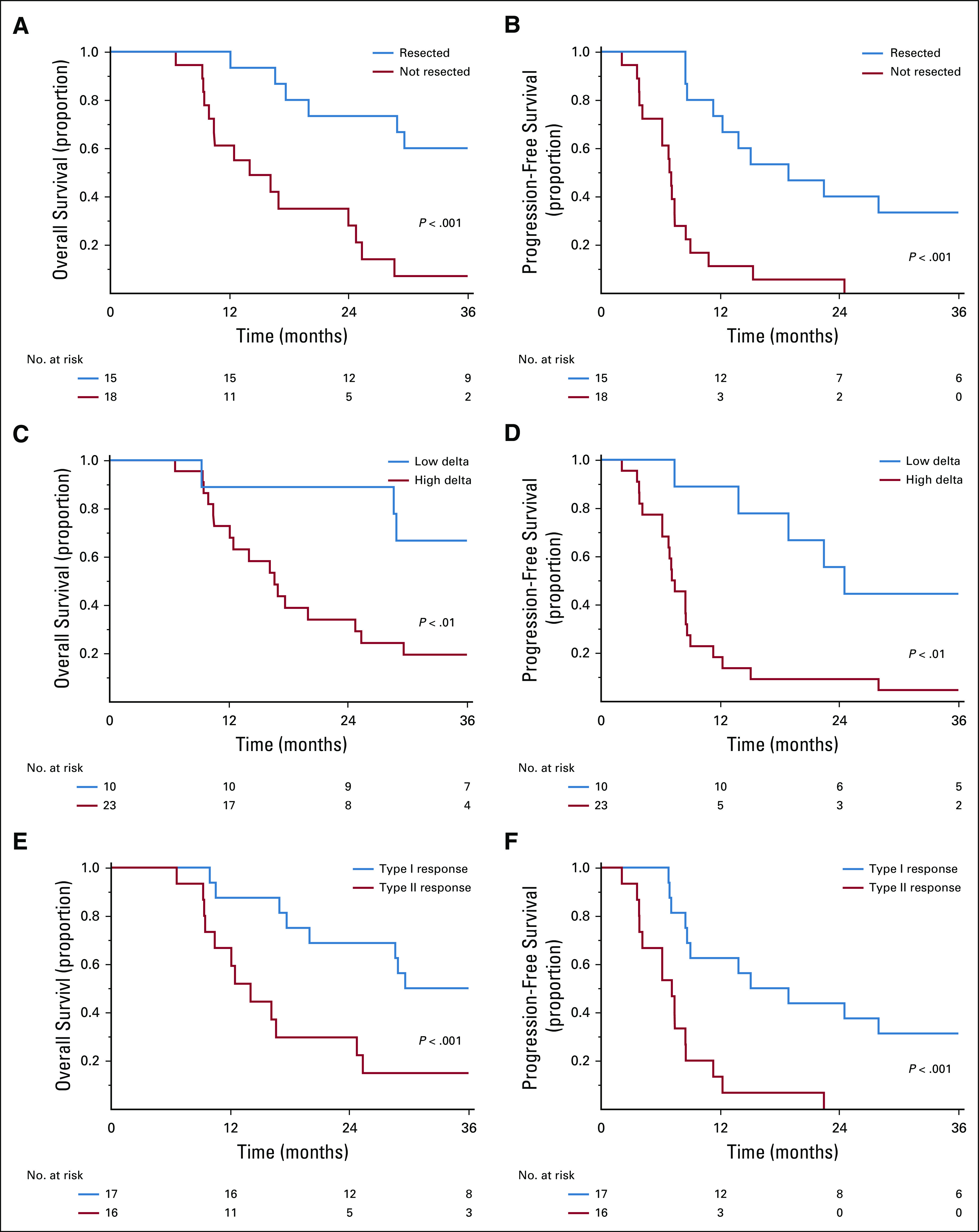
Kaplan-Meier estimates for (A) overall survival and (B) progression-free survival for resected (n = 15) and unresected patients (n = 18); (C) overall survival and (D) progression-free survival by delta classification; and (E) overall survival and (F) progression-free survival by interface response type.

The median OS of all 33 patients was 24 months (95% CI, 16.2 to 29.6 months); the median PFS was 8.7 months (95% CI, 7.2 to 13.9 months). The median OS duration was 42.1 (95% CI, 17.7 to not estimable) months and 14 (95% CI, 9.0 to 24.8) months for the patients who did and did not undergo pancreatectomy on protocol, respectively (Fig 3A). Patients who underwent pancreatectomy had longer median PFS (19 months; 95% CI, 8.7 months to not reached), compared with those who did not (7.0 months; 95% CI, 4.2 to 7.5 months; Fig 3B).

The median OS of patients with high-delta tumors was 17 months (95% CI, 12.0 to 25.0 months), whereas patients with low-delta tumors did not reach the median OS (95% CI, 9.3 months to not estimable; Fig 3C). The median PFS of patients with high-delta tumors was 7.5 months (95% CI, 6.2 to 8.7 months), compared with 23.5 months for those with low-delta tumors (95% CI, 7.4 months to not estimable; Fig 3D). Patients with high-delta tumors had significantly lower 3-year PFS (4% *v* 40%) and 3-year OS rates (20% *v* 60%) than those with low-delta tumors (both *P* < .05).

The median OS of patients with type I interface response was 30 months (95% CI, 18 months to not evaluable), compared with 14 months for patients with type II response (95% CI, 10 to 25 months; Fig 3E). The median PFS of patients with type I interface response was 15 months (95% CI, 8.5 months to not evaluable), compared with 7.3 months for those with type II response (95% CI, 3.9 to 8.6 months; Fig 3F). Patients with a type II interface response had significantly lower 3-year PFS (0% *v* 29%) and 3-year OS rates (16% *v* 47%) than those with type I response (both *P* < .001).

## DISCUSSION

This report adds to the recently published prospective experience with modified FOLFIRINOX delivered in a preoperative setting to patients with localized PDAC and provides prospective data to support the prognostic value of two biomarkers obtained from standard-of-care CT scans: delta score and interface response.

Several conclusions can be drawn from the results of this trial. First, the findings align with our previous observation that preoperative treatment sequencing accurately discriminates patients who are likely to achieve a survival benefit from surgery from those who are not.^19^ The median OS of the 15 resected patients was 42.1 months, and five patients remain alive without evidence of disease recurrence. The median OS of all patients, 24 months, was similar to that of the Alliance for Clinical Trials in Oncology (ALLIANCE) A021101 trial (ClinicalTrials.gov identifier: NCT01821612).^30^ From the baseline and operative characteristics (median CA 19-9 of 200 and ≥ 500 in eight patients; 48% of patients with both arterial and venous involvement, and a high rate of vein resections/reconstructions [14 of 15 resected tumors]), it is clear that this study population was enriched with cancers that were both anatomically and biologically advanced. In comparison, a single-institution phase II trial of total neoadjuvant therapy with FOLFIRINOX and personalized radiation for borderline resectable PDAC (median CA 19-9 of 97.5; 25% of patients with both arterial and venous involvement) reported a median survival of 37.7 months.^31^

Another important point involves patient selection. Although preoperative treatment sequencing helps identify patients at high risk for disease progression despite pancreatectomy, patients least likely to benefit from surgery cannot be identified prospectively before treatment. We have provided evidence that a baseline imaging-based delta classification can identify a priori the patients at high risk for metastatic progression during preoperative therapy. Furthermore, post-therapy, the interface response may further discriminate prognostic groups. Our data suggest that these imaging-based biomarkers outperform baseline CA 19-9 values in terms of prognostic value (Appendix Fig A3). Indeed, we have previously found baseline CA 19-9 values to not be predictive of outcomes,^2^ but have observed that changes in CA 19-9 are meaningful.^32^ The scientific basis of the delta classification and interface response seems to involve associations with degrees of stromal infiltrate, biologic drivers of the disease, and pathologic response to therapy.^8,9^ A combined approach of baseline and post-treatment imaging-based biomarkers using an early interim look may help inform preoperative and adaptive treatment recommendations for medical and surgical therapies, given both the demonstrated prognostic associations of the imaging-based biomarkers and the association of margin status with interface response. An advantage of our proposed imaging-based biomarkers is that they integrate into standard-of-care treatment. Although positron emission tomography–CT has shown promise as a diagnostic and prognostic marker for PDAC, it has inherent limitations with false negatives (eg, cold tumors), false positives (eg, pancreatitis), and higher cost.^33-37^

**FIG A4.**
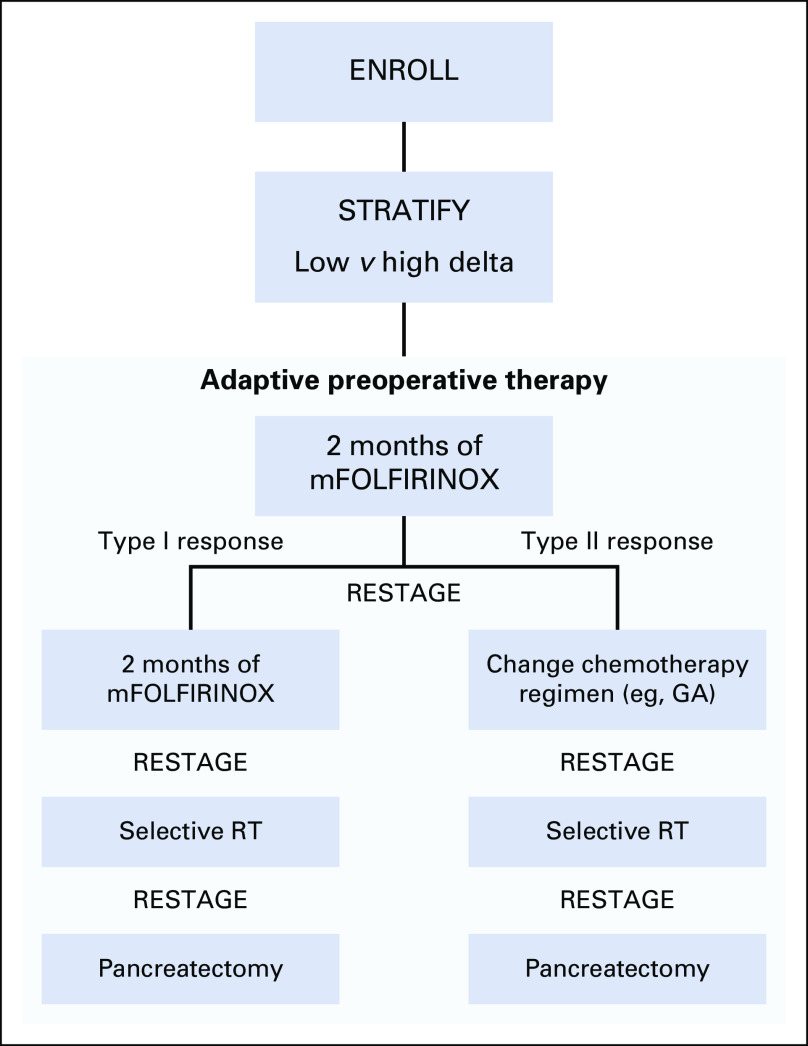
A trial concept for adaptive therapy using imaging-based biomarkers: patients would be stratified at baseline using high/low delta classification from imaging. After initiating mFOLFIRINOX (fluorouracil, leucovorin, irinotecan, oxaliplatin), patients would have restaging to assess imaging-based response (type I *v* type II). Patients who show a type II response would change chemotherapy, while those who have a type I response would continue with mFOLIFIRNOX. Selective radiation therapy (RT) would be delivered or not delivered based on clinical and anatomical factors. Pancreatectomy would be performed subsequently. If at any point, patients show metastatic progression, second-line chemotherapy would be given and the subsequent treatments in the diagram would not be done.

Our trial used aggressive cytotoxic regimens to treat patients with unfavorable clinical features without regard to the CT-based biomarkers. The data show that this unselected approach to therapy leads to rates of radiographic partial response (23%) within the range of previous studies (12% to 44%).^31,38^ Although this approach had reasonable toxicity rates (Table 2), the rates of resection were not as favorable as anticipated. Furthermore, given the high rate of distant metastasis during the administration of preoperative therapy, the role of a lengthy course of radiotherapy warrants reevaluation. The ALLIANCE protocol A021501 (ClinicalTrials.gov identifier: NCT02839343) will evaluate the role of hypofractionated radiotherapy in the preoperative setting for patients with borderline resectable disease who were selected only by failure to experience progression on induction chemotherapy.^39^ However, treatment intensification or selection for new combination systemic agents may be most appropriately considered in patients with high-delta tumors or type II responses. Our results argue for a more selective, personalized approach to preoperative therapy for patients with borderline resectable disease, driven by biomarkers (Appendix Fig A4).

Our study also highlights the need for rational and relevant end points for a borderline resectable trial that can be translated to a target population. Commonly cited end points include response rate, percentage of patients undergoing resection (the primary objective of this study), and rate of margin-negative resection. However, most of these features do not adequately predict relapse or OS. A positive study with a high resection rate could still have an OS in resected and unresected patients that may not measure up to success. In planning prospective trials for borderline resectable pancreas cancer, our results indicate that resection rate alone as a primary end point is inadequate. Until additional validation of our response readout is available, OS and disease-free survival need to be considered as coprimary or primary end points.

In conclusion, our data suggest that modified FOLFIRINOX followed by chemoradiation has a resection rate similar to historical controls for patients with borderline resectable PDAC. Our data indicate that the novel imaging-based delta classification and interface response of PDAC warrant additional investigation as predictive biomarkers for surgical benefit. The results also emphasize the need for new systemic agents and personalized approaches to push the therapeutic envelope for localized disease.

## Figures and Tables

**TABLE A1.**
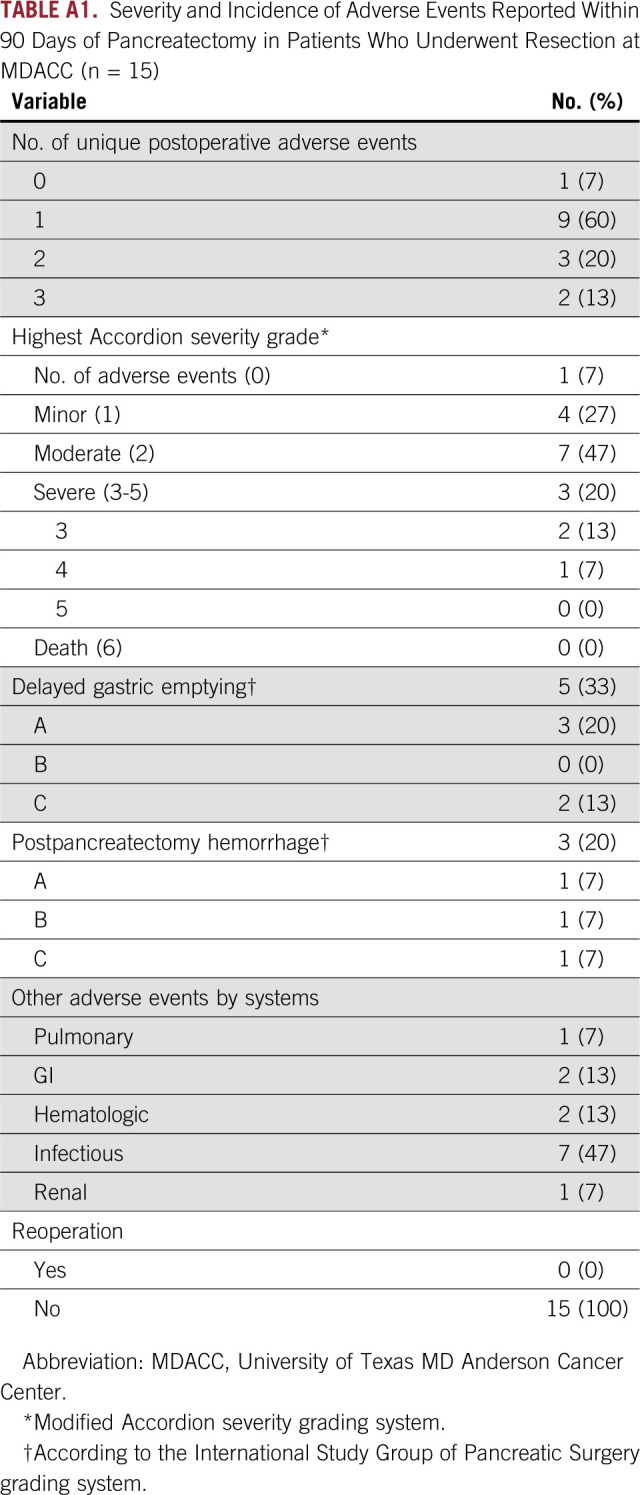
Severity and Incidence of Adverse Events Reported Within 90 Days of Pancreatectomy in Patients Who Underwent Resection at MDACC (n = 15)
